# The Hospital Isolation of Infectious Diseases

**Published:** 1913-08-09

**Authors:** H. Franklin Parsons

**Affiliations:** Late of the Medical Department, Local Government Board.


### THE HOSPITAL ISOLATION OF INFECTIOUS DISEASES.*

### III.—Recent Methods of Isolation and Nursing.

By H. FRANKLIN PARSONS, M.D., D.P.H., late of the Medical Department, Local Government Board.

In the last article we considered the difficulties and drawbacks which attend the hospital isolation of scarlet fever, and have led some to doubt whether the attempt to control the prevalence of the disease by this means is on the whole beneficial or worth the cost which it entails. But objections have in the past been raised to the hospital isolation of other diseases, and have been found removable by improved methods based on increased knowledge. Thus the dangers which were believed fifty years ago to arise from the aggregation of typhus cases in special wards ceased when ample distance between beds and cubic space, with free ventilation, were provided.

### Objections Met by Improved Methods.

The observed tendency of smallpox to spread around hospitals in which many acute cases of that disease were under treatment, which led Sir G. Buchanan in his evidence before the Hospitals Commission in 1882 to doubt whether the Asylums Board Hospitals, with their many excellences, had diminished the prevalence of smallpox in London, has been obviated by the removal of the hospitals to situations remote from populous places, and the disease has now ceased to be endemic in London.

Diphtheria, notwithstanding its admission to isolation hospitals, increased rapidly in the early nineties of the last century, reaching its maximum in 1893; but the introduction, about 1895, of the methods of bacteriological examination and serum injection removed many of the difficulties of diagnosis and treatment; and, as these methods have come into general use, the mortality from diphtheria has steadily fallen.

Similarly we may hope and expect that the progress of knowledge will remove the difficulties connected with the hospital treatment of scarlet fever. Of these the main one is the want of some definite and ready method of recognising the specific contagion of the disease, such as may afford a means of deciding the diagnosis in doubtful cases, and of determining when a convalescent patient has ceased to be infectious. In the meantime, however, an important advance is being made on the lines of what is called "bed-isolation." Although the question of what is the essential specific microbe of scarlet fever cannot be regarded as being yet decisively settled, it is becoming clear from the researches of Dr. Mervyn Gordon and others that the primary organism, whatever it may be, is commonly accompanied by other secondary pathogenic organisms, such as septic streptococci; and that it is to these superadded infections rather than to the original infection that the unfavourable features of severe cases and the supervention of complications are due. Moreover, these secondary infections may differ in different cases of scarlet fever. These mixed infections probably occur in other diseases also. It has long been known that there is a close association between scarlet fever and diphtheria; the one disease often supervenes upon the other; a scarlet fever convalescent sometimes gives rise to a return case of diphtheria and *vice versa*; and diphtheria bacilli are found in the throats of many patients who present only the symptoms of scarlet fever. From these facts it follows that persons suffering from scarlet fever should be isolated not only from healthy persons, but also in such a manner as to avoid the spread of secondary infections to or from other persons suffering from scarlet fever. As Dr. L. Parkes says:† "If the child sent to a fever hospital, whether with scarlet fever or diphtheria, can be isolated in his ward to the extent that he is not exposed to infection from his neighbours—either the infection of the disease from which he is not suffering (scarlet fever or diphtheria, as the case may be), or the more virulent form of infection of the disease from which he is suffering, which may very well happen under existing circumstances to be communicated to him—a great advance will have been made in securing true isolation in institutions which are now isolation hospitals in a somewhat limited sense. The existing fever hospital is certainly 'isolation' so far as the great body of the public is concerned, but is in no sense isolation of the sufferer amongst his fellow-sufferers."

### The Advance in Bed Isolation and Nursing.

Since these words were written considerable advance in securing isolation in this stricter sense has been made along two lines—viz., first, the provision of small single-bed wards or cubicles for the isolation of doubtful, mixed, or septic cases; and, secondly, aseptic nursing.

The nature and extent of the separation necessary between cases in order to prevent cross infection will depend upon the particular infection against which it is desired to guard, and its power of propagating itself through the air—its "striking distance." If the disease is spread only by more or less direct contact with persons or infected articles aseptic methods of nursing may suffice, but if it is spread through the air for any such distance as that between bed and bed in the same ward a physical barrier will be necessary. The opinions of authorities differ as to how far infection can be propagated through the air, and some are disposed to discredit aerial infection altogether, maintaining that the transference of infection can be sufficiently accounted for by direct contact. It is known, however, that in infectious diseases such as scarlet fever, measles, and diphtheria droplets of infected mucus from the mouth, throat, nostrils, and respiratory passages are liable to be expelled into

* Previous articles appeared on June 21 and July 19.
† *Public Health*, June, 1903.

the air by such acts as coughing, sneezing, or even speaking. Experiments have shown that bacilli may be carried through the air for distances up to seventy feet from the mouth of a speaker. Experience at certain hospitals has led to the conclusion that smallpox, chicken-pox, measles in the acute stage, and, in less degree, septic cases of scarlet fever cannot safely be treated in the same ward with cases of other diseases, even with the aid of aseptic nursing, and require to be treated in separate rooms.

### Types of Separation Wards.

Various forms of small "separation" wards have been devised for the isolation of cases in which a second infection is known or suspected to be present. Sometimes there are single-bed wards opening out of the main wards. In several recent hospitals the ward block has been divided into a number of compartments or cubicles by means of glazed partitions, the advantage of this arrangement being that the patients are more under the observation of the nurse, and feel less lonely when they can see each other. These compartments may be arranged on three different systems:—

(1) Each compartment is completely partitioned off from any other, and is separately entered from the open air under a verandah. (Examples may be seen at the Walthamstow, Croydon Borough, and Croydon Rural Hospitals.)

(2) Each compartment is completely partitioned off from any other, but is entered from a common corridor. (Examples may be seen at the East Ham Hospital, and at the Eastern Hospital of the Metropolitan Asylums Board.)

(3) The compartments (cubicles) are partially separated from one another by glazed partitions not reaching the ceiling. (Examples may be seen at the South-Western Hospital of the Metropolitan Asylums Board.)

Whatever the form of separation ward adopted, careful technique in nursing is an essential condition of success; and it is held by some medical superintendents that it may suffice, by itself and without any physical barrier, to prevent cross-infection in almost every case.

But it is evident that, even if the possibility of infection through the air can be excluded, to avoid all possible means of cross-infection between patients treated in a common ward requires a large amount of care and assiduity on the part of a numerous and highly trained staff of nurses, and where this condition cannot be ensured it will be better to put patients who are dangerous to one another into separate rooms.

### Dr. Cameron's Experiment.

A trial of the treatment of scarlet fever on aseptic lines in common wards made by Dr. A. F. Cameron in 1910-11 at the South-Eastern Hospital of the Metropolitan Asylums Board gave distinctly promising results as regards the avoidance of cross-infections and complications, and also as regards the percentage of cases which proved infecting after discharge.

One of the most important precautions for avoiding the transference of disease from patient to patient in a ward through the agency of droplet-infection is the maintenance of an ample distance between bed and bed. This distance should not be less than twelve feet, and in some instances fifteen feet has been allowed per bed. But, unfortunately, this distance is apt to be encroached on in times of pressure by putting more beds into the ward, so that the patients lie closer together, and the transference of infection from one to another by droplets expelled in coughing, or even by actual contact, is facilitated. This is probably one factor in producing the injurious effects of overcrowding in hospital wards.

Other methods which have been employed with more or less success for reducing the risk of cross-infection in hospitals and of persistent infectiveness after discharge are the careful inquiry as to the patient's antecedents and the risks of infection to which he may have been exposed; the keeping him in an observation ward until the nature of the illness is definitely ascertained; the anointing of his skin with eucalyptus oil; the open-air treatment of septic cases; the removal of the patient when the acute stage of his illness is over to a separate convalescent ward, and the detaining him after his final bath for a night in a discharge ward, so that he may not be exposed to risk of catching cold by returning home immediately after the bath. More attention is now paid to the state of the throat and mucous membranes and to the presence of a discharge from the nostrils or ears, as indicating whether the patient is still infectious, than to the peeling of the skin.

### Advantages of Joint Hospitals.

But the carrying out of modern aseptic methods of nursing requires an adequate staff of skilled nurses, and there is often a difficulty in securing such a staff at small hospitals which may often be without patients. This is one of the reasons why a combination of districts for the provision of a joint hospital of convenient size is preferable to the establishment of a small separate hospital for each district. Another means which has been suggested for overcoming the difficulty is the establishment of a nurses' exchange, by which nurses might be lent from one hospital to another according to the varying requirements of each. Such an exchange has been in operation for some years in the counties of Gloucester and Worcester with a certain amount of success.

The directions indicated in this article seem to me those in which we are likely to obtain the advantages of hospital isolation of infectious cases with the minimum of drawbacks.

